# Flavonoid Intake in Relation to Colorectal Cancer Risk and Blood Bacterial DNA

**DOI:** 10.3390/nu14214516

**Published:** 2022-10-27

**Authors:** Michela Carola Speciani, Marcello Cintolo, Mirko Marino, Maya Oren, Federica Fiori, Giorgio Gargari, Patrizia Riso, Clorinda Ciafardini, Federica Mascaretti, Maria Parpinel, Aldo Airoldi, Marcello Vangeli, Pierfrancesco Leone, Paolo Cantù, Pagona Lagiou, Cristian Del Bo’, Maurizio Vecchi, Pietro Carnevali, Barbara Oreggia, Simone Guglielmetti, Rossella Bonzi, Giulia Bonato, Monica Ferraroni, Carlo La Vecchia, Roberto Penagini, Massimiliano Mutignani, Marta Rossi

**Affiliations:** 1 Department of Clinical Sciences and Community Health, Branch of Medical Statistics, Biometry and Epidemiology “G.A. Maccacaro”, Università degli Studi di Milano, 20133 Milan, Italy; 2 Digestive and Interventional Endoscopy Unit, Azienda Socio Sanitaria Territoriale (ASST) Grande Ospedale Metropolitano Niguarda, 20162 Milan, Italy; 3 Department of Food, Environmental and Nutritional Sciences (DeFENS), Università Degli Studi di Milano, 20133 Milan, Italy; 4 Department of Medicine, University of Udine, 33100 Udine, Italy; 5 Gastroenterology and Endoscopy Unit, Fondazione Istituto di Ricovero e Cura a Carattere Scientifico (IRCCS), Ca’ Granda Ospedale Maggiore Policlinico, 20122 Milan, Italy; 6 Hepatology and Gastroenterology Unit, Azienda Socio Sanitaria Territoriale (ASST) Grande Ospedale Metropolitano Niguarda, 20162 Milan, Italy; 7 General Surgery Unit, Fondazione Istituto di Ricovero e Cura a Carattere Scientifico (IRCCS), Ca’ Granda Ospedale Maggiore Policlinico, 20122 Milan, Italy; 8 Gastroenterology and Digestive Endoscopy Unit, Fondazione Istituto di Ricovero e Cura a Carattere Scientifico (IRCCS), Istituto Nazionale Tumori, 20133 Milan, Italy; 9 Department of Hygiene, Epidemiology and Medical Statistics, School of Medicine, National and Kapodistrian University of Athens, GR-115 27 Athens, Greece; 10 Department of Epidemiology, Harvard T.H. Chan School of Public Health, Boston, MA 02115, USA; 11 Department of Pathophysiology and Transplantation, University of Milan, 20133 Milan, Italy; 12 Division of Minimally–Invasive Surgical Oncology, Niguarda Cancer Center, Azienda Socio Sanitaria Territoriale (ASST) Grande Ospedale Metropolitano Niguarda, 20133 Milan, Italy; 13 Fondazione Istituto di Ricovero e Cura a Carattere Scientifico (IRCCS), Ca’ Granda Ospedale Maggiore Policlinico, 20122 Milan, Italy

**Keywords:** blood, microbiome, flavonoids, anthocyanidin, flavanone, colorectal cancer, 16S rRNA gene profiling

## Abstract

Flavonoids have been inversely associated to colorectal cancer (CRC) and are plausible intermediaries for the relation among gut microbiome, intestinal permeability and CRC. We analyzed the relation of flavonoid intake with CRC and blood bacterial DNA. We conducted a case–control study in Italy involving 100 incident CRC cases and 200 controls. A valid and reproducible food–frequency questionnaire was used to assess dietary habits and to estimate six flavonoid subclass intakes. We applied qPCR and 16S rRNA gene profiling to assess blood bacterial DNA. We used multiple logistic regression to derive odds ratios (ORs) of CRC and Mann–Whitney and chi-–square tests to evaluate abundance and prevalence of operational taxonomic units (OTUs) according to flavonoid intakes. Inverse associations with CRC were found for anthocyanidins (OR for the highest versus the lowest tertile = 0.24, 95% confidence interval, CI = 0.11–0.52) and flavanones (OR = 0.18, 95% CI = 0.08–0.42). We found different abundance and prevalence according to anthocyanidin and flavanone intake for OTUs referring to Oligoflexales order, Diplorickettsiaceae family, *Staphylococcus*, *Brevundimonas*, *Pelomonas* and *Escherischia*–*Shigella* genera, and *Flavobacterium* and *Legionella* species. The study provides evidence to a protective effect of dietary anthocyanidins and flavanones on CRC and suggests an influence of flavonoids on blood bacterial DNA, possibly through intestinal permeability changes.

## 1. Introduction

The interplay between diet, gut microbiome, intestinal permeability and colorectal cancer (CRC) is receiving increasing interest and flavonoid intakes are plausible intermediaries for their relation [1,2,3].

Flavonoids are a group of polyphenols, characterized by a common structure of two aromatic rings bound together by three carbon atoms forming an oxygenated heterocycle [4]. They are commonly found in plant–based food and divided into six major subclasses (i.e., isoflavones, anthocyanidins, flavanols, flavanones, flavones, and flavonols) [4]. They have been demonstrated to act on pathways involved in cellular transformation, proliferation and apoptosis, as well as in angiogenesis and metastatic dissemination [5,6]. Moreover, they are known for their antioxidant and anti–inflammatory properties [7]. Local and systemic inflammation, as well as oxidative stress, are contributors to the development and progression of colorectal cancer [8]. The inverse association between dietary flavonoids and colorectal cancer risk has been also reported in various epidemiological studies [9,10,11].

Flavonoids can affect gut microbiota by exerting both prebiotic–like and antimicrobial activities and possibly leading to a selection of some species [12,13]. Particularly, glycosylated flavonoids (e.g., quercetin and kaempferol among flavonols, and hesperidin and narirutin among flavanones) are usually not absorbed in the small intestine and can be metabolized by colonic microorganisms [4]. Gut microbiota, in turn, has a relevant role in intestinal health and colorectal cancer risk [14,15]. Fecal metagenomic shotgun analyses show specific microbial signatures for CRC [1,16]. Among others, *Fusobacterium nucleatum*, *Bacteroides fragilis* and *Escherichia coli* have been positively associated to CRC [17].

Flavonoids have been also observed to have an impact on intestinal permeability both in vitro and in vivo [18,19,20,21,22,23]. Several mechanisms have been proposed (e.g., the regulation of the expression of tight, adherens and gap junctions, as well as desmosome proteins at a cellular level) to explain their capacity to counteract the passage of bacterial DNA within systemic circulation [24]. In epidemiological studies on circulating bacterial DNA, Acetobacteraceae, Bacteroidaceae, Lachnospiraceae, Peptostreptococcaceae and Ruminococcaceae families, *Flavobacterium* and *Ruminococcus* genera, and *Bacteroides fragilis* have been associated to CRC [25,26,27].

In this study, we analyzed the relation of flavonoid intake with colorectal cancer risk and circulating bacterial DNA, using data from an Italian case–control study [26].

## 2. Methods

We analyzed data from a case–control study conducted between 2017 and 2019 in two university hospitals in the metropolitan area of Milan, Italy [26].

Subjects were enrolled among eligible 20–85 years old patients scheduled for a colonoscopy. Immunodeficiency, selected inflammatory diseases, liver/kidney/heart failure, reported previous cancer, recent hospitalization or colonoscopy, as well as any dietary modifications in the previous month, were among the exclusion criteria.

We recruited 100 incident and histologically confirmed CRC cases and 200 controls, of which 100 intestinal adenoma (IA) and 100 free from IA/CRC subjects (hereafter referred to as healthy subjects), both frequency–matched (1:1) to CRC cases by study center, sex and age (±5 years). The assignment group was defined by two pathologists who reviewed colonoscopies and performed histological examinations when needed. Sixty–two percent of the final 300 subjects were men and thirty–eight percent were women. Mean age was 67 (range: 31–85) for CRC and 66 (range: 26–85) for controls.

Among contacted eligible patients, subjects who agreed to participate signed a written consent while less than 2% refused to participate in the study. The protocol was approved by the ethical committees of the involved hospitals: Fondazione IRCCS Ca’ Granda Ospedale Maggiore Policlinico (No. 742–2017) and ASST Grande Ospedale Metropolitano Niguarda (No. 477–112016).

### 2.1. Interview

The questionnaire was administered by blinded trained interviewers and included data on socio–demographics, education, lifestyle habits, and anthropometric measures.

A reproducible [28] and valid [29] food frequency questionnaire (FFQ) was used to collect data on each patient’s usual diet before colonoscopy. The FFQ included 75 items on foods, food groups as well as a range of the most common Italian recipes, and 6 items on alcoholic beverage consumption.

Subjects were asked to indicate their average weekly frequency of consumption for each dietary item; intakes lower than once a week and higher than once a month were recorded as 0.5 per week.

Using available data on the flavonoid content [30,31,32] of each food and ingredient considered in our FFQ, we developed an ad hoc food composition database in terms of 6 subclasses of flavonoids (mg/100 g): isoflavones, anthocyanidins, flavan–3–ols, flavanones, flavones, and flavonols. For items including multiple recipes or foods, with multiple ingredients, flavonoid contents were calculated by percentage of weight contribution of each ingredient. Taking into account the portion size of each item, dietary information from FFQ was translated into an average daily intake of flavonoids for each subject. Similarly, total energy intake was computed using an Italian food composition database [33].

### 2.2. Blood Collection

Blood samples were collected in 7 mL EDTA tubes before the colonoscopy and immediately stored at −80 °C. One mL microvial for each subject was sent to Vaiomer SAS, Labège, France, for metagenomic analyses. Operators were blinded to group assignment and all samples were analyzed in the same experiment and with the same reagent batches and manipulator.

### 2.3. DNA Extraction, Quantitative Polymerase Chain Reaction (qPCR) Experiments and Sequencing of 16S rRNA Gene Amplicons

An optimized blood–specific technique was used by Vaiomer SAS to perform bacterial DNA quantification and sequencing reactions [26,34,35].

An aliquot of 0.25 mL of whole blood was used for DNA extraction, collected in 0.05 mL of final extraction volume. Pan-bacterial primers used to perform real–time PCR amplification target the V3–V4 hypervariable regions of the bacterial 16S rRNA gene: EUBF 5′–TCCTACGGGAGGCAGCAGT–3′ and EUBR 5′–GGACTACCAGGGTATCTAATCCTGTT–3′ [36]. These primers show 100% specificity (i.e., no eukaryotic, mitochondrial, or Archaea DNA) and high sensitivity (16S rRNA of more than 95% of bacteria in the Ribosomal Database Project database were amplified).

The abundance of the 16S rRNA gene in blood was computed by using qPCR in triplicate, normalized with a plasmid–based standard range. Results were expressed in number of copies of 16S rRNA gene per µl of blood. MiSeq Illumina technology for 16S rRNA gene taxonomic profiling was then applied to DNA. The threshold of 5000 reads was not reached in four samples (2 IA and 2 healthy subjects), which were excluded from taxonomic profiling analyses [26].

Bacterial compositions were achieved by Vaiomer bioinformatic pipeline. The pipeline is comprehensive of the FROGS v1.4.0 operational taxonomic units (OTUs) algorithm of clustering and the Blast+ v2.2.30 to obtain the taxonomic assignment using Silva 132 Parc database. The reads obtained from the sequencing were demultiplexed, trimmed, and overlapped. The high–quality long reads were clustered removing the non–specific amplicons. Cluster analysis based on 97% sequence similarities using two steps (with an aggregation distance equal to 1 and equal to 3) of the swarm algorithm v2.1.6, was applied to identify OTUs. OTUs overpassing the relative abundance threshold of 0.005% of the whole dataset of reads were discarded. The raw reads are publicly available in the European Nucleotide Archive (ENA). The accession number is PRJEB46474.

Observed, Chao1, Shannon, Simpson and Inversed Simpson alpha–diversity indices were also calculated by R PhyloSeq v1.14.0 package, to assess DNA bacterial diversity in terms of richness and evenness in blood.

Potential bacterial DNA contamination from reagents or environment was assessed through the inclusion of several negative controls. Background noise and blood contamination did not impact the metagenomic analyses.

### 2.4. Statistical Analyses

We computed tertiles of intakes of each class of flavonoids and total flavonoids among controls. We applied logistic regression models, conditioned on the matching variable and further adjusted for education, energy intake, alcohol intake and body mass index to estimate odds ratios (ORs) of CRC and the corresponding 95% confidence intervals (CIs), using the lowest tertile of intake as a reference. Flavonoids were also entered as continuous variables for a measurement unit equal to one standard deviation computed among controls. Tests for trend were based on the likelihood ratio test between models with and without a linear term for each class of flavonoids. Additional adjustments for tobacco smoking, vegetable and fruit consumption were also considered.

Moreover, we computed the ORs of colon and rectal cancers separately. Stratified analyses were carried out according to sex, age (<70 and ≥70) and alcohol intake (<12 and ≥12 g/day).

We compared the distributions of 16S rRNA gene copies, selected alpha–diversity indices (Observed, Chao, Shannon, Simpson and Inversed Simpson) and relative abundance of the OTUs with a representation of at least 15 subjects (~5% of our sample) between subjects in the first two and in the third tertiles of anthocyanidin and flavanone intakes using a two–tailed Mann–Whitney test. We also evaluated differences in terms of presence or absence of OTUs according to tertiles using the chi–square test.

## 3. Results

Table 1 shows the distribution of CRC and controls according to selected characteristics. Controls tended to be more educated and to have a lower alcoholic consumption compared to CRC cases.

Table 2 gives the distribution of subjects, the ORs of CRC and the corresponding 95% CIs, according to the tertiles of the intake of flavonoid subclasses and total flavonoids. Total flavonoids were not related to CRC risk; the OR of the highest versus the lowest tertile was 0.65 (95% CI = 0.34–1.26). However, a significant inverse association with CRC risk was found for anthocyanidins and flavanones. The ORs were 0.24 (95% CI = 0.11–0.52) for anthocyanidins and 0.18 (95% CI = 0.08–0.42) for flavanones, both with a significant trend (*p* < 0.001). The OR for an increment equal to a standard deviation was 0.71 (95% CI = 0.51–0.98) for anthocyanidins and 0.42 (95% CI = 0.27–0.65) for flavanones. The ORs for the second and the third compared to the first tertile of flavones were 0.43 (95% CI = 0.22–0.87) and 0.93 (95% CI = 0.48–1.81), respectively, with a non–significant trend (*p* = 0.66); the continuous OR was 1.00 (95% CI = 0.78–1.27). Further adjustment for selected potential confounders gave similar results (data not shown). 

Table 3 gives the distribution of colon and rectal cancers, the corresponding ORs and 95% CI according to anthocyanidins and flavanones intakes. The ORs of colon cancer for the highest versus the lowest tertile were 0.32 (95% CI = 0.11–0.95; *p* for trend = 0.03) for anthocyanidins and 0.22 (95% CI = 0.07–0.67; *p* for trend = 0.01) for flavanones. The ORs of rectal cancer were 0.16 (95% CI = 0.05–0.52) for anthocyanidins and 0.12 (95% CI = 0.03–0.47) for flavanones, both with a significant trend (*p* < 0.001).

Associations between flavonoids and CRC risk were consistent among strata of sex, age and alcohol intake (Supplementary Table S1).

Table 4 shows the distributions of 16S rRNA gene copies according to the lowest two and the highest tertiles of anthocyanidin and flavanone intakes. The median (I–III quartiles) of gene copies was 7214.6 (5628.05–9663.55) in the lowest tertiles and 7141.9 (5642.10–8790.69) in highest tertile of anthocyanidin intake, and 7080.07 (5633.44–9212.02) in the lowest tertiles and 7724.91 (5634.11–9966.56) in highest tertile of flavanone intake. No significant differences were found in terms of gene copies according to tertiles of anthocyanidin and flavanone intakes (*p* = 0.769 and *p* = 0.346, respectively). Results were similar when the analysis was restricted to controls only.

In terms of alpha–diversity indices, subjects in the highest as compared to the lowest two tertiles of anthocyanidin intake had lower Shannon (*p* = 0.061) and lower Inversed Simpson (*p* = 0.047) indices, while subjects in the highest flavanone intake had a lower Simpson (*p* = 0.047). No relevant differences were found in terms of Observed and Chao indices, according to tertiles of anthocyanidin and flavanone intakes (data not shown).

Table 5 shows the relative abundance and/or prevalence of OTUs that differed in the first and second tertiles as compared to the third tertile of anthocyanidin and flavanone intake (*p* < 0.05). Subjects in the highest tertile of anthocyanidins had lower relative abundance and prevalence of the OTUs referring to *Flavobacterium* sp. (*p* = 0.001 and *p* = 0.009, respectively) and *Legionella* sp. (*p* = 0.015 and *p* = 0.020, respectively) as compared to the first two tertiles. Subjects in the highest tertile also presented a lower relative abundance of the OTUs belonging to the *Escherichia–Shigella* taxonomic group (*p* = 0.023) and higher prevalence of the OTUs belonging to the family of uncultured bacteria 0319–6G20 of the Oligoflexales order (*p* = 0.043). Subjects in the highest tertile of flavanones showed lower relative abundance and prevalence of the OTUs referring to *Flavobacterium* sp. (*p* = 0.002 and *p* = 0.008, respectively), *Legionella* sp. (*p* = 0.011 and *p* = 0.012, respectively), and belonging to *Staphylococcus* genus (both *p* = 0.017). Subjects in the third tertile also showed a lower prevalence of the OTUs belonging to the family Diplorickettsiaceae (*p* = 0.049), a higher prevalence of the OTUs belonging to *Pelomonas* genus (*p* = 0.013) and higher relative abundance and prevalence of the OTUs belonging to *Brevundimonas* genus (*p* = 0.013 and *p* = 0.016, respectively). These results were also confirmed after restricting the analysis to the group of controls for *Flavobacterium* sp. and *Legionella* sp., and for OTUs belonging to the family of uncultured bacteria 0319–6G20 for anthocyanidins, and for OTUs belonging to the genera *Brevundimonas* and *Pelomonas* for flavanones (data not shown). 

## 4. Discussion

This study suggests that higher intakes of dietary anthocyanidins and flavanones reduce the risk of CRC, with consistent results among strata of sex, age, alcohol intake and cancer location. The flavonoid subclasses were also shown to be linked to a different composition of blood bacterial DNA. In particular, anthocyanidins and flavanones were inversely related with OTUs referring to *Flavobacterium* sp. and *Legionella* sp., and positively related with the OTU referring to *Brevundimonas* genus. Anthocyanidins were also inversely related with the OTU referring to the *Escherichia—Shigella* genus group and positively related with the OTU referring to Oligoflexales order, while flavanones were inversely related with the OTU referring to Diplorickettsiaceae family and *Staphylococcus* genus and directly related with the OTU referring to *Pelomonas* genus.

Anthocyanidins have been associated to a reduced risk of CRC in a meta–analysis on five subclasses of dietary flavonoids and CRC. For anthocyanidin intake, that study considered seven studies (four case–control and three prospective studies) for a total of 13,023 cases, reporting an overall OR of 0.78 (95% CI = 0.64–0.95) for the highest versus the lowest category of intake [9]. Furthermore, the Southern Community Cohort Study (SCCS), involving 71,599 participants (787 incident cases) from the southeastern United States showed a hazard ratio (HR) of CRC of 0.78 (95% CI = 0.61–1.00) for the highest versus the lowest quintile of anthocyanidin intake [10].

Epidemiological results on flavanones are controversial. The previously mentioned meta–analysis selected ten studies (six case–control and four prospective studies) on flavanones, for a total of 14,833 cases and 618,512 controls and found a non–significant association with CRC risk (OR = 0.91, 95% CI = 0.71–1.17) [9]. The SCCS found similar results with an HR of CRC of 0.94 (95% IC = 0.75–1.18) for the highest versus the lowest quintile of flavanones intake.

Other flavonoid subclasses have been associated to a reduced risk of CRC, although not in our data, including isoflavones, especially in Asian populations, flavonols and flavones [9,37]. Isoflavones mainly derive from soy and soy products that are not part of the dietary tradition of our study population, possibly explaining our result.

In our population, anthocyanidins mainly came from the consumption of red fruits and wine, and flavanones from citrus fruit. Adjustment for fruit and vegetable consumption did not materially change the association we found between flavonoids and CRC, suggesting that these subclasses of flavonoids can explain, at least in part, the protective effects of a diet rich in plant foods on this cancer [38,39].

The supplementation with anthocyanin–rich bilberry extract prevented the formation and the growth of CRC in a mouse model [40]. Anthocyanidins’ anti–cancer biological activities could be mediated by various mechanisms including suppression of NF–κB signaling and activation of the Nrf2 and PI3K/AKT/survivin pathways, implicated in the regulation of colon cancer cells apoptosis and survival [41,42].

Various studies have described an in vitro anti–CRC activity of flavanones such as naringin, liquiritigenin and eriodictyol [43,44,45]. Naringin has been suggested to affect CRC through NF–κB/IL–6/STAT3, PI3K/AKT/mTOR, apoptosis, NF–κB–COX–2–iNOS and β–catenin pathways [43]. Liquiritigenin has been shown to affect HCT116 CRC cell line downregulating the expression of Runx2 and inactivating the PI3K/AKT pathway [44]. Eriodictyol seems to affect CRC cells by suppressing fucosylation and downregulating STAT3 expression [45].

Moreover, flavonoids can be involved in the modulation of intestinal permeability, a condition that can represent a critical factor for CRC risk [2,26]. Indeed, our data show that higher anthocyanidin and flavanone intakes are associated with a reduction of evenness indices of microbiota composition, which reflects a different passage of bacteria into blood through the intestinal barrier, especially considering rare bacteria abundances. No previous studies have investigated this issue, but a few recent intervention studies on another dietary component, i.e., fiber, mainly coming from food sources similar to those of flavonoids, reported decreased alpha–diversity indices in fecal samples upon dietary fiber treatment [46].

Both anthocyanidins and flavanones can interact with gut microbiota and positively impact inflammation and health [20,47,48,49]. Circulating *Flavobacterium* DNA has been inversely related with CRC in a Chinese study [27]. In line with our results, the abundance of *Flavobacterium* genus in gut microbiota was found to be higher in patients with multiple sclerosis [50] and with rheumatoid arthritis [51], both chronic inflammatory diseases, as compared to healthy controls. In preclinical studies, *Legionella* species have been associated with the activation of NLRC4 inflammasome, which has been implicated in CRC pathogenesis [52]. Moreover, a study on blood and stool microbiome found a higher proportion of *Legionella* genus in the blood of patients with IgA nephropathy, as compared to healthy controls [53]. That study also found higher *Escherichia–Shigella* bacteria in the stools of patients with IgA nephropathy, and higher *Staphylococcus* genus in the blood of patients with IgA nephropathy and reduced estimated glomerular filtration rate, as compared to healthy controls [53]. An in vitro study also suggested that anthocyanins may inhibit the growth of *Escherichia*–*Shigella* [54], and specific strains of intestinal *E. coli* have been positively associated with CRC risk [17,55]. *Pelomonas* was among the most important variables to discriminate CRC from adenomas and healthy subjects in a previous investigation on these data [26].

Weaknesses of our study are inherent to the case–control study design [56]. Selection bias cannot be excluded. However, we conducted an ad–hoc data collection using standardized procedures, which were fully observed by recruitment centers. By study design our cases and controls were comparable in terms of study center, sex and age, and were interviewed in similar settings.

A strength is that we collected subjects with a diagnosis of IA, which is a part of the pathogenetic sequence of CRC. In this investigation, we combined healthy and IA subjects as controls in order to increase the study power. However, when considering only healthy subjects as the control group, results were virtually identical. Moreover, no differences between IA and healthy subjects were found in terms of flavonoid intakes.

Another strength is that our cases were detected at the first CRC–diagnosing colonoscopy and were thus truly incident, reducing possible recent habit modifications due to the awareness of cancer diagnosis, without however excluding possible changes due to symptoms. Moreover, recall bias was considerably reduced, since they were unaware of their cancer diagnosis at the time of interview. We were able to further control information bias since interviewers and investigators were blinded to group assignment.

Possible bacterial contamination during colonoscopy was avoided by collecting all blood samples before the procedure, and we took care to analyze all blood samples in the same experiment with the same manipulator and reagent batches, aiming to keep an optimal signal-to-noise ratio and reduce technical variability. Some of the associations may be attributed to multiple testing.

Another limitation is the lack of stool samples which could have allowed us to estimate fecal microbiota and further investigate the interaction of flavonoids with intestinal bacteria and translocation.

The FFQ was satisfactorily reproducible [28] and valid [29] and was further integrated with questions aimed to evaluate dietary intakes, such as those of fiber and flavonoids, which have been related to changes in microbiota [3,57]. The FFQ was administered by trained interviewers, reducing misinterpretation and incorrect compiling. Imprecisions of exposure measurement due to different seasonality of data collection and to variability in food quantities in the recipes or in plant flavonoid content should be considered [4].

With reference to confounding, we adjusted for study center, sex and age, as well as for other possible confounders including education, energy intake, BMI and alcohol consumption. Moreover, further controlling for relevant OTUs in the models did not change the associations of anthocyanidins and flavanones with CRC risk (data not shown).

In conclusion, this research corroborates the preventive effect of anthocyanidin intake on CRC and brings evidence that flavanones may reduce CRC risk. Moreover, this is, to our knowledge, the first study to investigate flavonoids in relation to blood bacterial DNA, suggesting a possible interplay among diet, gut microbiota, intestinal permeability and genetic bacterial material in blood.

## Figures and Tables

**Table 1:** Distribution of 100 colorectal cancer (CRC) and 200 controls * by selected characteristics. Italy 2017–2019.

Characteristic	CRC	Controls
**Study center**		
Niguarda	65 (65.0%)	130 (65.0%)
Policlinico	35 (35.0%)	70 (35.0%)
**Sex**		
Male	62 (62.0%)	124 (62.0%)
Female	38 (38.0%)	76 (38.0%)
**Age group**		
<50	10 (10.0%)	11 (5.5%)
50–59	19 (19.0%)	43 (21.5%)
60–69	29 (29.0%)	62 (31.0%)
70–79	31 (31.0%)	62 (31.0%)
≥80	11 (11.0%)	22 (11.0%)
Mean (SD) age (years) ^†^	66.1 (11.6)	65.9 (11.3)
**Education (years) ^‡^**		
<7	25 (25.0%)	31 (15.5%)
7–11	25 (25.0%)	50 (25.0%)
≥12	49 (49.0%)	119 (59.5%)
**Energy intake ^§^ (kcal/day)**		
<1589	35 (35.0%)	66 (33.0%)
1589–2003	30 (30.0%)	67 (33.5%)
≥2004	35 (35.0%)	67 (33.5%)
**Alcohol intake ^§^ (g/day)**		
<2.65	28 (28.0%)	65 (32.5%)
2.65–14.31	29 (29.0%)	68 (34.0%)
≥14.32	43 (43.0%)	67 (33.5%)

* 100 intestinal adenoma and 100 free from intestinal adenoma/CRC subjects. ^†^ *p* value of the *t*–test for heterogeneity = 0.9. ^‡^ The sum does not add up to the total because of one missing value. ^§^ Tertiles computed among controls. SD: standard deviation.

**Table 2:** Odds ratios (OR) * of colorectal cancer and 95% confidence intervals (CI) for flavonoid intakes among 100 cases and 200 controls. Italy 2017–2019.

Classes of Flavonoids (mg/Day)	Mean (SD) ^†^		Tertiles ^†^		$\chi_{1}^{2}$ (*p*–Value)	Continuous ^‡^
		I	II	III		
**Isoflavones**	32.1 (40.5)					
Upper cutpoint		4.93	35.02	–		
CRC : controls		35 : 66	22 : 68	43 : 66		
OR (95% CI)		1	0.54 (0.26–1.13)	1.20 (0.65–2.20)	0.80 (0.37)	1.23 (0.96–1.58)
**Anthocyanidins**	28.3 (32.3)					
Upper cutpoint		9.45	30.62	–		
CRC : controls		49 : 66	38 : 67	13 : 67		
OR (95% CI)		1	0.69 (0.38–1.23)	0.24 (0.11–0.52)	12.30 (<0.001)	0.71 (0.51–0.98)
**Flavan–3–ols**	25.1 (40.3)					
Upper cutpoint		8.39	21.37	–		
CRC : controls		34 : 67	31 : 66	35 : 67		
OR (95% CI)		1	0.98 (0.51–1.89)	0.88 (0.42–1.84)	0.13 (0.72)	0.96 (0.74–1.25)
**Flavanones**	20.3 (23.0)					
Upper cutpoint		3.96	24.01	–		
CRC : controls		46 : 67	45 : 66	9 : 67		
OR (95% CI)		1	0.90 (0.51–1.61)	0.18 (0.08–0.42)	14.30 (<0.001)	0.42 (0.27–0.65)
**Flavones**	0.45 (0.23)					
Upper cutpoint		0.35	0.49	–		
CRC : controls		45 : 67	20 : 66	35 : 67		
OR (95% CI)		1	0.43 (0.22–0.87)	0.93 (0.48–1.81)	0.19 (0.66)	1.00 (0.78–1.27)
**Flavonols**	29.0 (16.5)					
Upper cutpoint		18.90	33.90	–		
CRC : controls		32 : 67	33 : 66	35 : 67		
OR (95% CI)		1	1.06 (0.57–1.98)	1.17 (0.61–2.24)	0.31 (0.58)	1.21 (0.94–1.54)
**Total Flavonoids**	135.2 (85.9)					
Upper cutpoint		89.44	155.40	–		
CRC : controls		37 : 66	37 : 68	26 : 66		
OR (95% CI)		1	0.98 (0.54–1.76)	0.65 (0.34–1.26)	1.03 (0.31)	0.89 (0.68–1.16)

* Estimates from logistic regression model conditioned on study center, sex and age, and adjusted for education, energy intake, alcohol intake and BMI. ^†^ Computed among controls. ^‡^ OR estimated for an increment equal to a standard deviation (computed among controls).

**Table 3:** Odds ratios (OR) * of colon cancer and rectal cancer and 95% confidence intervals (CI) among 50 colon and 50 rectal cancer cases and 200 controls. Italy 2017–2019.

		Tertiles ^†^		$\chi_{1}^{2}$ (*p*–Value)	Continuous ^‡^
	I	II	III		
**Colon cancer**					
** Anthocyanidins**					
Cases	25	18	7		
OR (95% CI)	1	0.62 (0.27–1.46)	0.32 (0.11–0.95)	4.79 (0.03)	0.82 (0.54–1.25)
** Flavanones**					
Cases	24	20	6		
OR (95% CI)	1	0.62 (0.27–1.42)	0.22 (0.07–0.67)	7.04 (0.01)	0.42 (0.22–0.80)
**Rectal cancer**					
** Anthocyanidins**					
Cases	24	20	6		
OR (95% CI)	1	0.64 (0.27–1.50)	0.16 (0.05–0.52)	8.19 (<0.001)	0.59 (0.35–0.99)
** Flavanones**					
Cases	22	25	3		
OR (95% CI)	1	1.11 (0.47–2.62)	0.12 (0.03–0.47)	8.35 (<0.001)	0.39 (0.21–0.73)

* Estimates from logistic regression model conditioned on study center, sex and age, and adjusted for education, energy intake, alcohol intake and BMI. ^†^ Computed among controls. ^‡^ OR estimated for an increment equal to a standard deviation (computed among controls).

**Table 4:** Distribution of 16S rRNA gene copies in whole blood/µL, according to tertiles of anthocyanidin and flavanone intakes, overall and among controls. Italy 2017–2019.

	Tertiles *		Mann Whitney *p*–Value
	I–II	III	
**Anthocyanidins**			
** Overall**			
N.	220	80	
Median (I–III quartiles)	7214.6 (5628.1–9663.6)	7141.9 (5642.1–8790.7)	0.769
** Among controls**			
N.	133	67	
Median (I–III quartiles)	7104.2 (5683.6–9104.2)	7218.2 (5638.8–8769.8)	0.772
**Flavanones**			
** Overall**			
N.	224	76	
Median (I–III quartiles)	7080.1 (5633.4–9212.0)	7724.91(5634.1–9966.6)	0.346
** Among controls**			
N.	133	67	
Median (I–III quartiles)	7056.7 (5655.0–8338.5)	7478.9 (5447.2–10290.6)	0.323

* Computed among controls.

**Table 5:** Distributions of relative abundance and prevalence of selected operational taxonomic units (OTUs) in blood according to anthocyanidin and flavanone intake. Italy 2017–2019.

OTUs	Mean		Median (I–III Quartiles)		Mann Whitney *p*–Value	Prevalence (%)		$\chi_{1}^{2}$*p*–Value
	Tertiles		Tertiles *			Tertiles *		
	I–II	III	I–II	III		I–II	III	
**Anthocyanidins, n**	218	78	218	78		218	78	
p_Bacteroidetes;c_Bacteroidia;o_Flavobacteriales;f_Flavobacteriaceae;g_Flavobacterium;s_Flavobacterium sp.	2.180	1.144	0.011 (0.000–3.884) ^†^	0.002 (0.000–0.217)	0.001	158 (72.5) ^‡^	44 (56.4)	0.009
p_Proteobacteria;c_Deltaproteobacteria;o_Oligoflexales;f_0319–6G20;g_Unknown;s_Unknown	0.665	0.595	0.000 (0.000–0.003)	0.000 (0.000–0.005)	0.176	70 (32.1)	35 (44.9) ^‡^	0.043
p_Proteobacteria;c_Gammaproteobacteria;o_Enterobacteriales;f_Enterobacteriaceae;g_Escherichia–Shigella;s_Multi–affiliation	1.505	1.267	0.007 (0.002–2.353) ^†^	0.002 (0.000–0.367)	0.023	169 (77.5)	52 (66.7)	0.059
p_Proteobacteria;c_Gammaproteobacteria;o_Legionellales;f_Legionellaceae;g_Legionella;s_Legionella sp.	0.295	0.044	0.000 (0.000–0.000) ^†^	0.000 (0.000–0.000)	0.015	45 (20.6) ^‡^	7 (9.0)	0.020
**Flavanones, n**	221	75	221	75		221	75	
p_Bacteroidetes;c_Bacteroidia;o_Flavobacteriales;f_Flavobacteriaceae;g_Flavobacterium;s_Flavobacterium sp.	2.192	0.164	0.010 (0.000–4.059) ^†^	0.002 (0.0–0.521)	0.002	160 (72.4) ^‡^	42 (56)	0.008
p_Firmicutes;c_Bacilli;o_Bacillales;f_Staphylococcaceae;g_Staphylococcus;s_Multi–affiliation	0.144	1.297	0.000 (0.000–0.000) ^†^	0.000 (0.000–0.000)	0.017	16 (7.2) ^‡^	0 (0)	0.017
p_Proteobacteria;c_Alphaproteobacteria;o_Caulobacterales;f_Caulobacteraceae;g_Brevundimonas;s_Multi–affiliation	0.060	0.178	0.000 (0.000–0.000)	0.000 (0.000–0.000) ^†^	0.013	21 (9.5)	15 (20) ^‡^	0.016
p_Proteobacteria;c_Gammaproteobacteria;o_Betaproteobacteriales;f_Burkholderiaceae;g_Pelomonas;s_Multi–affiliation	3.422	4.315	2.645 (0.018–4.966)	3.676 (1.602–6.129) ^†^	0.013	206 (93.2)	74 (98.7)	0.071
p_Proteobacteria;c_Gammaproteobacteria;o_Diplorickettsiales;f_Diplorickettsiaceae;g_Unknown;s_Unknown	0.262	0.222	0.000 (0.000–0.000)	0.000 (0.000–0.000)	0.072	46 (20.8) ^‡^	8 (10.7)	0.049
p_Proteobacteria;c_Gammaproteobacteria;o_Legionellales;f_Legionellaceae;g_Legionella;s_Legionella sp.	0.280	0.057	0.000 (0.000–0.000) ^†^	0.000 (0.000–0.000)	0.011	46 (20.8) ^‡^	6 (8)	0.012

* Computed among controls. ^†^ Higher abundance. ^‡^ Higher prevalence.

## Data Availability

All the reads are publicly available in the European Nucleotide Archive (ENA) with the accession number: PRJEB46474.

## Supplementary Materials

The following supporting information can be downloaded at: https://www.mdpi.com/article/10.3390/nu14214516/s1, Table S1: Odds ratio (ORs) * of colorectal cancer and 95% confidence intervals (CIs) for anthocyanins and flavanones intakes among 100 cases and 200 controls, according to variables of interest. Italy 2017–2019.
