# Supplementary material for: Flavonoid Intake in Relation to Colorectal Cancer Risk and Blood Bacterial DNA
Source: Nutrients. 2022 Oct 27;14(21):4516. doi: 10.3390/nu14214516 (PMC9653960; doi:10.3390/nu14214516)
Supplement: Supplementary file 1 [file nutrients-14-04516-s001.zip › nutrients-1958106-supplementary.pdf]

Table S1. Odds ratio (ORs)* of colorectal cancer and 95% confidence intervals (CIs) for anthocyanins and flavanones intakes among 100 cases and 200 controls, according to variables of interest. Italy 2017-2019.

	**Anthocyanidins**			**Flavanones**		
	Tertiles†			Tertiles†		
	I	II	III	I	II	III
**Sex**						
Males						
CRC : Controls	33 : 40	21 : 39	8 : 45	28 : 39	29 : 42	5 : 43
OR (95% CI)	1	0.52 (0.23-1.15)	0.19 (0.06-0.54)	1	0.86 (0.4-1.83)	0.12 (0.04-0.42)
Females						
CRC : Controls	16 : 26	17 : 28	5 : 22	18 : 28	16 : 24	4 : 24
OR (95% CI)	1	0.79 (0.31-2.02)	0.27 (0.07-0.97)	1	1.18 (0.4-3.49)	0.19 (0.05-0.76)
**Age**						
<70						
CRC : Controls	28 : 37	21 : 43	9 : 36	25 : 38	27 : 38	6 : 40
OR (95% CI)	1	0.62 (0.29-1.30)	0.27 (0.09-0.75)	1	1.03 (0.49-2.14)	0.21 (0.07-0.63)
≥70						
CRC : Controls	21 : 29	17 : 24	4 : 31	21 : 29	18 : 28	3 : 27
OR (95% CI)	1	0.49 (0.15-1.57)	0.14 (0.04-0.54)	1	0.60 (0.21-1.76)	0.13 (0.03-0.55)
**Alcohol intake (g/day)**						
<12‡						
CRC : Controls	24 : 33	21 : 36	6 : 41	25 : 36	22 : 35	4 : 39
OR (95% CI)	1	0.85 (0.39-1.89)	0.21 (0.07-0.59)	1	0.92 (0.42-1.99)	0.15 (0.05-0.5)
≥12‡						
CRC : Controls	25 : 33	17 : 31	7 : 26	21 : 31	23 : 31	5 : 28
OR (95% CI)	1	0.6 (0.27-1.33)	0.3 (0.1-0.89)	1	0.99 (0.44-2.22)	0.25 (0.07-0.84)

* Estimates from logistic regression model conditioned on study center, sex and age, and adjusted for education, energy intake, alcohol intake and BMI.

† Computed among controls.

‡ Amount corresponding to one standard alcoholic drink.
